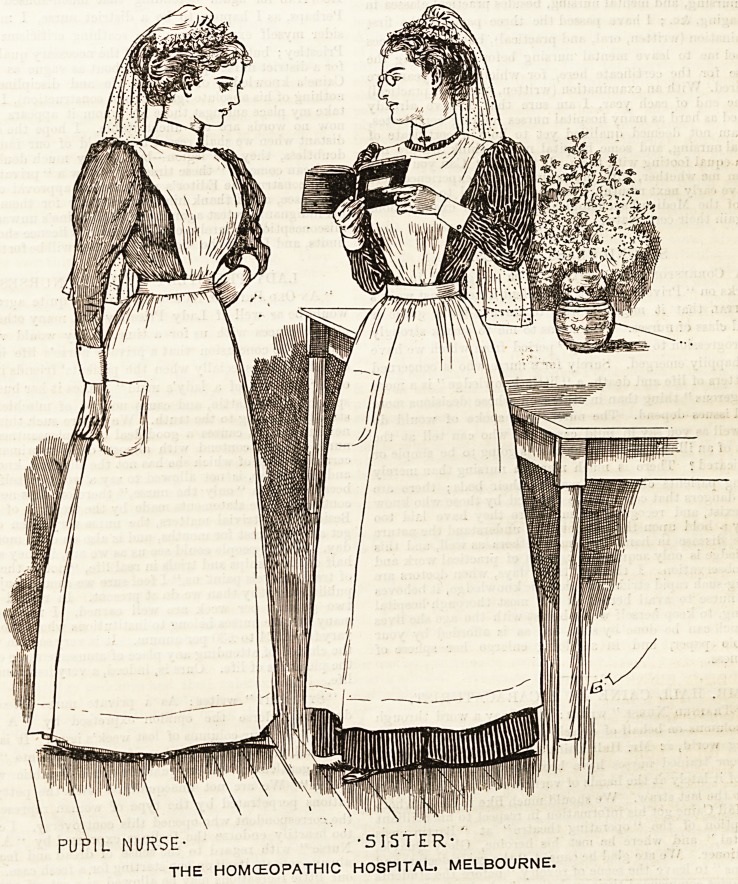# "The Hospital" Nursing Mirror

**Published:** 1897-02-06

**Authors:** 


### "The Hospital" Nursing Mirror.

Being the Nursing Section of "The Hospital."

[Contributions for this Section of "The Hospital" should be addressed to the Editor, The Hospital, 28 & 29, Southampton Street, Strand, London, W.C., and should have the word "Nursing" plainly written in left-hand top corner of the envelope.]

### News from the Nursing World.

### H.R.H. PRINCESS CHRISTIAN AT CHELSEA.

H.R.H. Princess Christian was present on Tuesday at an At Home given by Miss de Pledge at the Nurses' Home, Chelsea Infirmary. The Princess on her arrival was presented with a very pretty bouquet by Miss de Pledge. Her Royal Highness went over the whole building before leaving, and expressed much admiration at the completeness and comfort of all the arrangements. It is certainly a charming home, and looked its very best on Tuesday. During the afternoon an excellent concert was given in the nurses' recreation-room by Miss Lucy Clarke and other artistes, and in a mysterious little alcove fortune-telling provided much amusement. Amongst the guests were Sir Dyce and Lady Duckworth, Sir James Crichton Browne, Mr. and Mrs. Langton, Mr. Mark Hovell, Dr. Bezly Thorne, Dr. and Mrs. Louis Parkes, and Mr. Douglas Gordon.

### THE QUEEN'S REIGN.

At a meeting of the Town Council of Windsor last Saturday (January 30th) it was decided to call a public meeting to consider the steps to be taken to commemorate the present year, and to recommend to the meeting that after providing for "public rejoicing" the surplus funds subscribed should be divided equally between the Windsor Royal Infirmary and Princess Christian's District Nurses' Home, the latter institution having been established in the year of the Jubilee.—A Congregational minister, the Rev. J. Chadburn, of Sutton, Surrey, has offered to give £5,000 towards the endowment of a ward for cancer patients at the New Hospital for Women, Euston Road, if another similar sum be forthcoming. — Mr. Passmore Edwards has offered £2,000 in aid of a new cottage hospit al at Acton, on condition that a site is found for the building and a local fund started for its endowment.

### ROYALTIES AND CHARITIES.

H.R.H. the Princess of Wales has consented to become a patroness of the Free Home for the Dying at Clapham. The Duchess of Sutherland is president of the Home.—The Senior Physician of the Ventnor Consumption Hospital has received a letter from Lord William Cecil, Equerry in Waiting to the Queen, telling "how exceedingly pleased" Princess Henry of Battenberg was with all she saw on her visit to the hospital last week. The Princess has consented to lay the foundation-stone of he new block at the end of July this year.

### NURSES ON WHEELS.

Following the good example set at Guy's Hospital a Nurses' Bicycling Club has been started by the Lady Superintendent and the two lady medical officers at the Claybury Asylum, and some machines have been procured upon which the beginners may learn.

### ST. THOMAS'S HOSPITAL NURSES' CONCERT.

A very large audience filled the hall in the treasurer's house at St. Thomas's on the occasion of the nurses' concert last week. Many of the honorary medical staff were present, and numbers of "Old Nightingales," as well as a proportion of the present sisters and nurses. The programme was excellent, Madame Belle Cole singing four songs, whilst Miss Elsie Mackenzie, Mr. Norman Salmond, and the Meister Glee Singers also kindly gave their services. Dr. Toller's violin solos were a real musical treat, and were loudly applauded. Mr. and Mrs. Upton gave an amusing scenette, and Mr. Tebbutt sang two solos.

### THE NURSE'S VEIL.

A correspondent to the *Westminster Gazette* asks some pertinent questions with regard to the "veil of mystery" attached to the headgear of sick nurses remarking that "it is not worn over the face, but careers wildly in the wind, getting in the way of passers-by . . . . a sort of lasso, at times winding round the nurse's neck, then flying out (presumably laden with hospital microbes) to smite and wind round the necks of other people." Evidently the writer has had the misfortune to walk on a windy day within reach of a particularly obnoxious specimen of that sort of veil mostly affected by private nurses. Hospitals, for the most part, do not require their nurses to wear an expensive and useless piece of frippery, which, if it is to look at all well, needs constant renewal, costs a good deal, and really serves no purpose except to get in its wearer's way and make her needlessly conspicuous. The veil is, of course, merely a relic of the conventual habit and veil, handed down by tradition from days of old. It is far better absent from the bonnet of the practical trained nurse of to-day.

### ANOTHER NURSE FOR POOLE.

Lady Wimborne takes a great deal of interest in nursing matters in her neighbourhood, and, besides her support of the Cornelia Hospital, has for some years maintained a district nurse in Poole at her own expense. Lady Wimborne is now appealing to the people of Poole to come forward with the wherewithal to establish a second nurse, one being found quite insufficient for so large a district. There must be many in and around Poole who should help with money and personal interest in carrying through this necessary scheme, and Lady Wimborne ought not to ask in vain for their support.

### DIFFICULTIES AT SHEFFIELD.

In consequence of certain serious charges made against the nurses at the Children's Hospital at Firvale, the whole question of the management of that institution has come up for the consideration of the Sheffield Board of Guardians. The committee appointed to inquire into the charges reported that they considered them proved, and suggested the appointment of a permanent charge nurse, at the same time recommending that the nursing staff be under the direct authority of the superintendent and the matron of the children's homes. To this report one of the Guardians objected, and made some very sensible remarks on the undesirability of placing the nurse under the sole orders of an untrained superintendent, for all who had experience of hospital work knew the difficulties which arose when persons without special training attempted to interfere with those who had it. He contended that the infirmary superintendent was the proper person to be in charge of the Children's Hospital. Ultimately the matter was referred back to the Hospital Committee.

### CONWAY DISTRICT NURSING ASSOCIATION.

NURSE ROBERTS has had a heavy year's work in Conway. She herself paid some 3,006 visits during 1896, and at one time the services of a second nurse became necessary. The report presented at the recent annual meeting showed that the association was doing well, and possessed a balance in hand of £46 12s. 1d. It was decided at the meeting to devote £10 to the Carnarvon county scheme of contribution to the Queen Victoria's Jubilee Institute Commemoration Fund.

### "NURSING NOTES" AND MISS NIGHTINGALE.

A GOOD suggestion is made in the February number of *Nursing Notes*. Hearing that the National Portrait Gallery contains no portrait of one whom English men and women must ever delight to honour—Florence Nightingale—the Editors propose that the nurses of England might well subscribe to pay for the execution of a copy of an excellent painting of Miss Nightingale, once exhibited at a military exhibition, to be added to our national portraits. The picture in question probably belongs to some member of Miss Nightingale's family, and would surely be gladly lent for such a purpose. Nurses wishing to express their approval of the idea should write to the Editor of *Nursing Notes*, 12, Buckingham Street, Strand.

### A SAD AFFAIR.

A SAD tragedy occurred on Monday evening at 43, York Street, Baker Street, when a young man named Thomas Arnold shot Miss Maud Waller, upon whom he had been calling, afterwards turning the revolver upon himself, with fatal result. Miss Waller has lately been on the stage, but had previously been for some time at the Bedford General Infirmary, where Arnold had come under her care as a patient. It is stated that she had refused to marry Arnold, and that it was a repetition of this refusal which led to the tragedy. Miss Waller was taken at once to King's College Hospital. It has not yet been found possible to extract the bullet.

### NORTHUMBERLAND COUNTY NURSING ASSOCIATION.

THIS association has just been formed on the lines of the Lincolnshire and other institutions of a similar kind. It is intended to employ "two classes of nurses," the first to be "Queen's Nurses," the second to have been trained for not less than six months in district nursing approved Q.V.J.I., and holding the certificate of the L.O.S. The Northumberland Technical Education Committee have promised four scholarships of £35 each to the new association, that sum defraying the cost of six months' training at the District Nurses' Home at Plaistow. Women applying to be trained under this system are required to give their services to the association for three years at a minimum wage of 12s. a week. The association makes affiliation with itself conditional upon compliance with the requirements of the Queen's Jubilee Institute, which has now undertaken the regular inspection of such county associations.

### MR. BANCROFT AT THE LONDON HOSPITAL.

MR. BANCROFT gave his reading of Dickens' "Christmas Carol," in aid of the funds of the London Hospital on Monday night, in the fine library of the Medical College—"*not* the operating theatre," as Mr. Holland explained—to the amusement of those among the audience who had read Mr. Hall Caine on hospital festivities. The library, which holds some six hundred people, was filled to overflowing with a very appreciative audience. Mr. Bancroft, in generously helping the hospitals, has given a great deal of pleasure to his hearers, for his rendering of the well-known Christmas story is a thing not to be forgotten. The Hon. Sydney Holland was in the chair, and amongst others present were Mr. J. H. Buxton, Dr. and Mrs. Gilbart Smith, Mr. Treves, and Dr. Stephen Mackenzie.

### PRINCE ALFRED HOSPITAL, SYDNEY.

THE annual examination at the Prince Alfred Training School for Nurses, Sydney, took place at the end of December. The questions set included papers on anatomy and physiology, invalid cookery and elementary nursing, for nurses in their first year; on medical and surgical nursing, the nursing of sick children, and materia medica for those in their second year; and for third-year candidates on massage and electricity, ophthalmic nursing, nursing of the insane, and gynæcological nursing. Besides the theoretical examination in invalid cookery, there was a practical demonstration. Sir Alfred Roberts (hon. secretary), and the medical superintendent of the Prince Alfred Hospital, inspected the dishes made and awarded the marks. Miss McGahey is much to be congratulated on the high standard of training which is maintained at this hospital, of which all who are connected with it have every reason to be proud. We shall give the examination questions in full another week.

### SHORT ITEMS.

THE committee of the Singer Ball, held at Coventry in November of last year, have handed £10 of the proceeds to the Coventry and District Nursing Association.—The Salisbury Board of Guardians have been considering the question of subscribing to the local district nursing associations. The matter has been referred to a committee, and the clerk instructed to obtain copies of the rules of the various associations for the further information of the Guardians.—A sad accident is reported from Birkenhead. Nurse Morris, of Bedington, fell from the platform at the Rock Ferry station in front of the London express and was terribly injured. She was taken at once to hospital.—An enjoyable entertainment was got up by Dr. E. W. Lewis lately for the patients and nurses of the West London Hospital.—Another assistant nurse has resigned at the City of London Union Infirmary on the ground, as explained to the Board by one of the lady guardians, that "it was impossible to work with the doctor, and that no lectures had been held for over fifteen months."—There are some interesting letters on "The Future of the Private Nurse" in *Nursing Notes* for February, which all private nurses will do well to read. The article on "Incidental Opportunities of District Nurses," in the same number, is excellent.—A "Children's Party" was held on January 30th at the West End Hospital for Paralysis, Welbeck Street.

### Pharmacy and Dispensing for Nurses.

By C. J. S. Thompson.

### II.—ALTERATIVES—ANÆSTHETICS—ANODYNES—ANTACIDS—ANTEMETICS—ANTHELMINTICS—ANTHIDROTICS—ANTIPARASITICS—ANTIPERIODICS AND ANTIPYRETICS.

Having considered some of the chief forms used for administering medicine, we will proceed to enumerate the principal remedial agents of definite operation, classed according to their therapeutic action.

Alteratives are medicines which gradually change and correct a morbid condition of the bodily organs. They include oxide, sulphurated, and tartarated antimony, arsenic and its preparations, dilute nitro-hydrochloric acid, ammonium chloride, potassium chlorate, sodium chloride, iodine and the iodides, phosphorus and the hypophosphites, and the salts of potassium. Of mercury, we have the mixture of mercury with chalk (grey powder), blue pill, calomel, and the perchloride. Of sulphur, the sublimed, precipitated, and the sulphides. Among vegetable bodies, dulcamara, guaiacum, hemidesmus (renal), sarsaparilla, mezereum, and dandelion, leptandrin, phytolaccin, podophyllin, rumicin, and sanguinarin.

Anæsthetics are substances which suspend consciousness, or cause insensibility to pain. They may be divided into two classes, viz.: (1) General, administered by inhalation; (2) local, administered by spray or other application to the part. In the first class we have ether, pure and methylated (it was first used as an anæsthetic for capital operations in 1846, and pure ether is still preferred by some to chloroform, as it has a much less depressing effect upon the heart, and may be used for prolonged operations); ethel bromide, which is very rapid in its action; the A. C. E. mixture, which is composed of chloroform, ether, and rectified spirit; and tetrachloride of carbon. Chloroform, more frequently used than any other anæsthetic, is obtained by distillation from a mixture of chlorinated lime, slaked lime, and weak spirit. Other anæsthetics of this class are methylene, introduced in 1867, nitrous oxide gas, chiefly used in dental operations, and Regnauld's anæsthetic mixture, which is composed of chloroform and methylic alcohol.

Among local anæsthetics we have acid carbolic, ether (spray), ethyl bromide, antipyrine (administered hypodermically), cocaine (now largely used for producing anæsthesia in the eye and portions of the mucous membrane), eucaine iodoform, menthol, and methyl chloride (which produces intense cold caused by its rapid evaporation).

Anodynes are medicines which alleviate pain by lessening the excitability of nerves or nerve centres. Among those of chemical origin are the bromides of potassium, ammonium, and sodium, all valuable anodynes, the two latter salts having a less depressing effect than the former; butyl chloral hydrate, chloral hydrate, chloroform, amyl nitrite (by inhalation, in angina pectoris, and asthma, and restorative in defective breathing). Among the more modern remedies we have antipyrine (its anodyne properties being especially useful in neuralgia and gout), antifebrin (in neuralgia and nerve affections), exalgin, and phenacetin. Anodynes of vegetable origin include aconite and aconitine (used in acute rheumatism and facial neuralgia), belladonna and atropine (the former given in nervous and inflammatory affections and the latter used locally to relieve pain), caffeine, cajepute oil, camphor, and Indian hemp. Opium, morphine, and codeine, conine, conium, and veratrine are all powerful anodynes. Of milder action we have henbane, hops, piscidia, scopola, stramonium, and spirit of ether.

Antacids are medicinal agents which correct acidity of the secretions, and naturally consist of alkaline bodies. Of these we may mention ammonia carbonate, aromatic spirit of ammonia, bismuth lozenges, lime water (useful in diarrhœa connected with acidity), carbonate of lime, prepared chalk, compound decoction of aloes, carbonate and citrate of lithia, magnesia and its carbonate (valuable in dyspepsia, heart burn, sick headache, gout, and other complaints attended with acidity), solution of potass, potassium carbonate and bicarbonate, sodium carbonate and bicarbonate, solution of soda, soda water, and hard soap.

Antemetics are medicines which arrest vomiting from disease, sea sickness, &c. Among these may be included carbolic acid (which is given in small doses from one to two grains to check sickness and flatulence), hydrocyanic acid (in combination with other drugs to allay vomiting), diluted phosphoric acid (useful in cases of vomiting arising from bilious attack), nitrate of silver, bismuth salts (which are extremely valuable in some forms of irritative dyspepsia accompanied by vomiting), solution of lime, oxalate of cerium (of great use in chronic vomiting), chloral hydrate, chloroform, hydrochlorate of cocaine (said to be successful as a preventive to sea-sickness in doses of a quarter to one grain), creasote (given to arrest nausea in hysteria, and for obstinate sea-sickness), mercury with chalk (chiefly given to children in diarrhœa and vomiting), bicarbonate of potass (usually prescribed for sickness, in effervescence with citric acid), effervescing potass and soda water, bicarbonate and bromide of soda, and oxide of zinc (which is given in spasmodic affections).

Anthelmintics (vermicides) are medicines given to destroy worms or expel them from the alimentary canal, and are thus termed vermifuges. The vermicides used for destroying ascarides or thread-worms are: carbolic acid, aloes, perchloride of iron, sulphate of iron, quassia, senna, chloride of sodium, castor oil, turpentine, and santonin. For round worms, santonin. For tape-worm: kousso, sodii santonas, liquid extract of male fern, pomegranate root and bark, pelletierine sulphate and tannate, kamala, and turpentine. Vermifuges include areca nut (for round and tape worm), calomel, gamboge, jalap, castor oil, and scammonium.

Anthidrotics are medicines which check perspiration. The following are frequently prescribed: Acetic acid diluted, salicylic acid, sulphuric acid diluted, tannic acid, agaricus (employed in the night sweating of phthisis), atropine (for excessive secretion from the sweat glands), belladonna, sulphate of iron, compound iron mixture, decoction of logwood, henbane, picrotoxine (as a remedy against profuse sweating), quinine, scopola, strychnine, and oxide of zinc.

Antiparasitics are agents used for destroying vegetable and animal parasites. They include sulphurous acid, a powerful deoxidising agent destructive to vegetable life; oleate of copper (employed in ringworm, when diluted with lard), ammoniated mercury (used for pediculi), iodine (employed externally for ringworm), naphthalene (a valuable insecticide, and used with success in scabies), pyrethrum flowers, quassia, hyposulphite of sodium (used as a lotion for parasitic skin diseases), sozoiodol (used as a local application for the same purpose), staphisagria (employed in the form of ointment as a non-irritating remedy in scabies and as a parasiticide), and sulphur, and tobacco as insecticides.

Antiperiodics are medicines which have the property of interrupting periodical attacks of disease. Of these the salts of quinine are most frequently used, and of great value in intermittent fevers, when given in large doses. They also include arsenious acid (prescribed in agues and neuralgic affections), sulphate of berberine, liquid extract of cinchona, bebeeru bark, salicin, and chloride of sodium.

Antipyretics are medicines which reduce the temperature in fever. Among the older remedies are aconite, tartarated antimony, citrate of potass, quinine, and salicin. Within the last few years this class of medicinal agents has been largely increased by a series of synthetic bodies, mostly derived from coal tar, which have become very popular with prescribers. These bodies are said to produce changes in the blood, diminishing its respiratory capacity and destroying red corpuscles. They include antifebrin, antipyrine, antisepsin, analgene, chinoline (given in doses of from three to ten drops), exalgin (dose, 2 to 6 grains), formanilide (dose, 1 to 4 grains), kairine (dose, 5 to 8 grains), neurodin (dose, 5 to 15 grains), phenacetin (dose, 5 to 10 grains), iodopyrin (dose, 5 to 20 grains), salipyrin (dose, 15 to 30 grains), tolysal (dose, 5 to 20 grains), phenocoll hydrochlorate (dose, 7 to 15 grains), salol, salocoll (dose, 10 to 30 grains), thalline sulphate (dose, 3 to 8 grains), and thermodin (dose, 5 to 15 grains).

### Post-Graduate Clinics for Nurses.

By a Trained Nurse.

### I.—INTRODUCTORY.

It is a common observation among private nurses, "I am getting quite rusty and old-fashioned. I do wish I could go back to Hospital for a time to smarten up. There are so many up-to-date wrinkles and such new developments in nursing since the days of my training that I do not feel nearly so valuable to doctor and patient as I should like to be."

This complaint on the part of nurses is undoubtedly based on a real difficulty, which presses heavily on those who wish to keep up with the times and raise their professional value; and this hunger for more knowledge prompts many a private nurse to re-enter Hospital for a time, and is in itself a very healthy indication, showing the nurse to be ambitious for her art and animated by a spirit of progression.

The rapid strides made in medicine bring in their train new developments of nursing, and there is an ever-increasing demand on the part of the medical man of to-day, both in Hospital and private practice, for more intelligent and helpful co-operation from his nurses. So that, if nursing is to go hand-in-hand with medical science, it follows as a matter of course that post-graduate instruction for nurses already certificated must be established. Indeed, this seems such an absolute essential that it is to be hoped the question will soon be dealt with in a practical manner.

The necessity for some such systematised instruction has long been recognised in the United States, and admirable facilities are given there for nurses who wish to specialise in their work after graduating in a general Hospital, or who feel the need of going occasionally "back to school," so as to become conversant with higher and newer branches of their art. I have often heard it stated by physicians in New York quite as an accepted axiom that "a private nurse whose certificate dates three years back is not so useful a help-meet to the practitioner—despite her longer experience—as is the new-fledged nurse fresh from the Training School, and primed with all the newest of new methods."

This feeling on the part of medical men, coupled with that spirit of "go aheadism" characteristic of our Trans-Atlantic neighbours, has, at least in many of the New York Hospitals, led to the establishment of post-graduate courses for nurses of the most valuable nature. At the Woman's Hospital the nursing of cases of gynæcology, operations, and the general care of women's diseases may be acquired in short courses by nurses already certificated; while at the Babies' Hospital the care of sick children may be added to a general training, and certificates in midwifery and obstetric nursing can be obtained by a nurse in New York without the expenditure which holds back many an English nurse from taking the L.O.S.

"I feel quite like an antique—a sort of nurse who came out of the ark," a graduate said recently who took her certificate quite brilliantly some five years ago at a leading London Training School, and has since been engaged in private nursing. Of course, hers was an exaggerated and fanciful method of stating matters; but a good deal of truth underlies the exaggeration. "In the days when I was a pro.," she continued, "poultices were used in pneumonia as a matter of routine. Now, the other day a doctor in a little village 15 miles from a railway station described poultices as antedeluvian and prehistoric. And, to add to my troubles, the doctor in a previous case asked me if I understood how to take care of a patient undergoing the 'perpetual bath' treatment. But the last straw was piled up when a general practitioner in Birmingham expected me, quite as a matter of course, to give electrical treatment to one of his patients. I had only once seen a battery, and was horribly afraid if I touched the wires that there would be a terrible explosion of something or other. It is no wonder I feel fossilised, and I have every intention of going back to hospital for three months in the guise of a humiliated and elementary 'guinea-pig.'"

On all hands the same complaints are being voiced, and it is to be regretted that this, the most practical and necessary of all nursing questions—the providing of a definite post-graduate course, which would enable the trained nurse to keep in touch with the later and newer methods of her Training School as these arise—is not being dealt with. To the great detriment of the profession all sorts of "grievances" of an imaginary nature are being discussed, and dissatisfaction and discontent aroused over mere trifles, while the establishment of post-graduate teaching—an innovation which would undoubtedly meet with the approval of the majority o modern nurses—"hangs fire" for the want of a little organisation by some leading spirits.

In the absence of such definite and practical training it is proposed in these clinics, from week to week, to bring before nurses engaged in hospitals and those employed in private work the advances which are being made in the various branches of medical and surgical nursing. It is hoped that these clinics will, in some measure, supply the place of that post-graduate teaching which the near future will assuredly see established, not only in London, but in all our large provincial centres where Training Schools exist.

The private nurse especially labours under the disadvantage of isolation in her work and from the lack of a stimulating competition. She therefore is in some danger of falling below a standard, a danger increased by the small opportunity she has of seeing others at work and thus keeping up to date in a professional sense. If she live in a nursing home she "picks up" a good deal from fellow nurses, who are perhaps newer from hospital than herself; but any knowledge she gains is acquired in scrappy and unsatisfactory fashion. Beside which it can hardly be expected—and it certainly would not be hygienic—that she should employ her leisure and brief intervals between cases in talking "shop," and finding out by a system of cross-examination of her fellow workers the recent additions to a nursing *repertoire*. It is true that private nurses in London have the advantage of attending occasional lectures and demonstrations in nursing, these often being of a very valuable nature. But the private nurse necessarily lives more or less "in her trunk," and must from the nature of her calling be a bird of passage, so that she can never count on attending a systematic course of lectures, however valuable and necessary to her work these may be. She can, therefore, only fit in an occasional lecture at those times when the convalescent condition of her patient or leisure between cases allows her some time off duty. At present, then, she can rely only on nursing literature to keep her in touch with new methods in the care of the sick, and, as most nursing books are written for the instruction of hospital workers, and few ever take into consideration the needs of the private nurse, her opportunities for learning by reading are limited. So far it seems hardly to have been recognised what an enormous gulf separates the private from the hospital worker, and how different are the needs of each, so that there has not been a sufficient differentiation of their position. In the course of these articles I shall hope to point out the many practical differences between the nursing of a patient in a hospital ward and the care of a sick person at home. I shall aim, too, at setting forth the latest and most approved methods in the nursing of each case dealt with, so that I hope my nurse-readers will be able to take a satisfactory post-graduate course "in print."

### A Few Practical Hints for New Probationers.

Experience in the ward training of probationers tends to show that there are certain errors common to all, and particular traps which ensnare the majority. Here are a few hints based upon a practical knowledge of some of the rocks of offence. First and foremost with regard to your equipment, O new probationer. Much depends upon your first appearance, and very often at a glance a sister will form an opinion as to the probability of your "doing" or not. If you are wise you will not arrive on duty in your last summer's fashionably cut cotton dress, with a few inches sweeping the ward floor. You will do better to invest in a plainly made workmanlike print of no very noticeable pattern or colour, the skirt just clearing the floor. Your cap and apron will probably be supplied by the hospital. Don't let your cap rest upon rough and tousled hair, and if you have a fringe dispense with it before you enter the doors of a hospital.

Be sure your shoes are comfortable and silent. Bring with you a sharp pair of surgical scissors and a pair of dressing forceps. On no account append from your waist a dangling, jingling châtelaine; avoid jewellery and all the outward paraphernalia of the modish nurse.

So much for personal appearance; now a word as to behaviour. An observance of hospital etiquette is very important, and is more insisted on in some hospitals and wards than in others. In the wards the sisters belong to the highest ranks professionally, and demand deference and respect from those working under them. The staff nurses are also office bearers, and, as such, they also require respect from their subordinates. Remember you can be subservient without being servile.

Don't argue. Even if you feel you have been misunderstood, let the matter rest; it will probably explain itself, and you will have gained more by silence than if you had wasted time and words in argument. Don't expect praise; be content not to be blamed, for it is the equivalent to praise in hospital.

Always finish your own work before offering to help others. Keep your eyes and ears open, and learn all you can by observation. Above all things, be thorough — slow and behind-hand at first you are sure to be, but from the moment you enter a ward you can be thorough. You will often be tempted to be quick at the expense of thoroughness, but never yield to it, for it does not pay in the long run. It will be an immense satisfaction at the end of your probation if you can feel you have never scamped your work. Remember that every detail is of consequence. Your work and method will be watched and criticised when you are least aware of it, and your reports will be based upon what is observed when and where you least expect observation.

Our training is no sinecure, and it never ends. It is a building up day by day of facts and knowledge by that stern instructor Experience, and we gradually learn the truth of the words:

"And other's follies teach us not,
Nor much their wisdom teaches;
And most of sterling worth is what
Our own experience preaches."

"Ryndon."

### A Club for Nurses.

We are happy to be able to announce that the Victoria Club for Nurses and Associated Workers was opened this week. The want of such an establishment conducted on strictly neutral lines has long been felt by town and country nurses, who often desire to meet their friends in comfortable and quiet quarters, or seek rest and refreshment for themselves. The Victoria Club has been designed to meet this want. It is admirably situated in The Hospital Building, Southampton Street, Strand. It contains handsome drawing, lunch, and tea-rooms, lecture, writing, and cloak-rooms, besides convenient offices. All are comfortably and tastefully fitted. The Building is lighted by electric light throughout, and all apartments are well warmed and airy. In the drawing-room a comprehensive selection of weekly and daily journals will be found, and in the entrance lobby a notice board is fixed containing the announcements of meetings, lectures, and other events likely to be of interest to members. The telephone with which the club is connected will be a great convenience to country and other members by facilitating arrangements for appointments and other business. Post graduate lectures will be given from time to time by able lecturers in quick succession, so as to give country members an opportunity of attending as many as possible during a short stay in London. The secretary, herself a trained nurse of varied experience, will be glad to afford all information and assistance in her power, and to aid members in securing suitable quarters when desired, a list of apartments being kept for their convenience. A comprehensive reference library of books dealing with hospitals and kindred subjects will shortly be opened in the Building. Thus, in the Victoria Club, nurses and others, for whose advantage it has been established, will find a most useful and pleasant centre, which will materially add to the comfort and interest of their professional life. The subscription for nurse members is only £1 1s., and for associated workers £2 2s. per annum. No entrance fee will be asked of members joining before June, 1897.

### Where to Go.

Children's Home Hospital, Barnet.—Two performances of "Sweet Lavender" will be given by well-known amateurs in aid of this useful little institution on Friday evening, February 19th, and Saturday afternoon, February 20th, at the Bijou Theatre, Westbourne Grove. Tickets, price 5s. and 2s. 6d., may be obtained from Mrs. Arthur Waugh, 11, Hillfield Road, West Hampstead.

Stafford House.—Mr. S. B. Bancroft has kindly consented to read, on behalf of the Chelsea Hospital for Women, Dickens's "Christmas Carol," on Tuesday afternoon, February 16th, at half-past three, at Stafford House, St. James's, mostly kindly lent for the occasion by the Duke and Duchess of Sutherland. This will be the last of this most interesting series of readings given by Mr. Bancroft this season, and the only matinée.

### The Canary Islands for Health or Pleasure.

THE choice of a spot in which to spend the winter depends mainly on four considerations, viz., the climate, the journey to and fro, the hotel accommodation, and the resources of the place. From the invalid's point of view, climate is by far the most important, since if that is unsuitable all other advantages are of no avail. It is pre-eminently from their climatic advantages that the Canaries are rising so rapidly into favour for winter sojourns. From October till April the weather in these Fortunate Isles presents a steady average of temperature not far removed from the perfection of comfort. A picked day out of an English June with cloudless sky, soft airs, and exhilarating sense of freshness will about equal the normal state of things during the day; but the delicious balmy evenings, which render it possible even for invalids to linger late out of doors, enjoying the warm, dry air, have no parallel at any time of the year in England.

In the two main places of resort, Las Palmas in Grand Canary, and Orotava in Teneriffe, certain minor differences of climate exist; each has its adherents, and most people try both before leaving the Islands. Las Palmas is a shade hotter; the sunshine is almost uninterrupted, and while an Englishman may sometimes find the heat in the middle of the day oppressive, even a shivering convalescent from the West Coast of Africa will admit "there is no frost in the air." The air is intensely dry, and many sufferers from diseases of the throat find it most restorative, and this in spite of the dust which makes walking an impossibility and driving a penance. There is a chilly period about sunset when invalids are obliged to be careful, but with this exception the regularity of the temperature makes it possible to be out of doors from morning till night.

At Orotava the climate is rendered remarkably equable by its position at the foot of the Cordilleras in an amphitheatre formed by these mountains sloping down to the sea. The following is the mean temperature Fahrenheit during the winter months: October, 69·3; November, 68; December, 66·7; January, 62·2; February, 62·1; March, 64·2. The mean winter temperature is 63·8, as compared with 61·7 at Madeira, 49·6 at Nice, and 41·7 in London. The air is very rich in ozone, and though more rain falls than in Las Palmas it falls mainly at night, and by reason of the porous properties of the soil is hardly perceptible an hour after. The resident physician, Dr. Victor Perez, who has had some years' experience at Brompton Consumptive Hospital, and bears a high reputation in the Island of Teneriffe, has found the climate to exercise a markedly beneficial effect in nearly all chest and bronchial complaints. Many sufferers from the after effects of influenza, depression, insomnia, &c., find the open-air life and the charm of the perpetual sunshine tempered by cool breezes of more avail than a multitude of drugs. In rheumatic affections, needless to say, the dry air is invaluable. Dr. Perez has found that in persons suffering from diseases of the kidney, diabetes, &c., albuminaria rapidly disappear. Many sufferers from anæmia, malaria, scrofula, joint disease, and gout have derived great benefit from the healthy conditions of life which accompany a stay at Orotava. The water is excellent, flowing from a spring out of the solid rock, and the place has shown unusual immunity from typhoid.

It is a marked feature both at Las Palmas and Orotava that though a good proportion of the visitors, as at most of these winter resorts, have come for the benefit of their health, the invalid element is by no means conspicuous, for nearly every one is on the road to health and is correspondingly cheerful. At both places several striking cures in long-standing cases came under the notice of the writer even during a few weeks' stay. The majority is made up of those in search of rest and change, or seeking to avoid the winter.

The voyage to Las Palmas by the Castle Line takes from four to five days, a return ticket costing £24. The British and African Steamship Company, however, have a good service running weekly from Liverpool at £15 return, and the Forwood Line runs a smaller service of steamers weekly from London at the same rates. The voyage by either of these lines lasts from eight to nine days, but after the first three days calm seas are the rule, and, except for very bad sailors, the extra time on board is not unpleasantly spent. The return ticket on both these lines permits the passenger to go on to Teneriffe and Madeira.

The hotel accommodation in the Canaries is good and moderate. The Metropole, at Las Palmas, situated on the sea-shore, has excellent rooms at from 8s. a day "en pension," including besides the usual meals, early morning and afternoon tea, and, if desired, beef-tea, &c., in the middle of the morning. This includes also the use of the well-appointed bath-rooms. The cooking and attendance is good, and the sanitary arrangements entirely modern and satisfactory.

The Catalina Hotel at Las Palmas bears also a good reputation, and has the additional advantage of a pretty garden. Those who find the heat excessive at any time can find a charming and bracing sojourn for a week or two at Quiney's Hotel at Monte, a lovely spot among the hills a few miles inland from Las Palmas.

In Teneriffe the principal hotel is the Taoro, or English Grand, at Orotava. It is reached by a drive of 26 miles from Santa Cruz, the only port of Teneriffe, and has an unrivalled situation in several acres of well-planted ground. An endless variety of tropical flowers and trees are interspersed with shady walks, where in hammock, deck-chair, or inviting summer-house, the visitors find themselves almost independent of indoor accommodation. From here a view is to be had of the snow-clad peak rising 12,000 feet from behind the Canadas or great snowy table-land in the distance; 300 feet below the hotel nestle the brown roofs of the little fishing village, and beyond, across the intense blue of the Atlantic may be seen the shadowy outline of the island of Palma. The Taora hotel has recently passed under English management, and is well appointed. The lowest terms are 12s. a day for single rooms, and 11s. a day where two share a room together. A lady nurse, of whose skill and attentive kindness all unite in speaking most highly, resides in the hotel, and her services are free to all who require them.

Several pensions in the neighbourhood of the Grand offer good accommodation at moderate terms. The hotels at the Puerto below, though less expensive, are in a less healthy situation, and cannot be recommended for invalids.

As regards the resources of the Canaries, it may be well to caution every intending sojourner to go provided with some kind of occupation. Any hobby will prove an invaluable blessing during the lazy, aimless days of hotel life. Tennis, golf, and croquetare, are the outdoor sports, and at Orotava good walking and riding "of sorts." The all-important bicycle will find no pasture in Grand Canary, but in Teneriffe the one finely-made "carretera" presents a beautiful surface across the island for about fifty miles. In Grand Canary there are about half-a-dozen stock drives to places of interest—mostly too far off for comfort—and the pleasure of any excursion is severely interfered with by the shocking condition in which the horses are often sent out.

At the leading hotels there are weekly dances and concerts during the season, and the usual amusement committees to help people to kill time.

A few words will be sufficient with regard to Madeira, whose attractions have been well before the public for many years. Its advantages in certain well-defined cases of lung disease are well known; but a doctor should be consulted before the effect of the warm, moist climate is tried. The luxuriant vegetation resulting from the abundant rainfall makes it a far more beautiful island than any of the Canaries; but those in good health are likely to find it enervating by comparison. The principal hotel, Reid's at Funchal, is famed for the beauty of its situation and the excellence of the management.

### Dress and Uniform.

By a Matron and Superintendent of Nurses.

### THE HOMŒOPATHIC HOSPITAL, MELBOURNE.

The Homœopathic Hospital holds its own as one of the three important hospitals at Melbourne. Judging by the smart appearance of its nursing staff we should imagine it had attained a high level of efficiency and had kept pace with all the advances which have been made in recent years. The head nurse of this hospital wears a costume of dark navy fastened with buttons on to the waistband. A tasteful cap of the coronet shape in white spotted muslin is worn on the head and the crown terminates in a long end reaching half-way down the back. Two rows of frilling edged with valenciennes lace make a becoming finish to the cap and soften the outline of the face. Linen cuffs and collar relieve the dress at the neck and wrists.

The pupil nurse, whom the illustration depicts on the left

PUPIL NURSE· ·SISTER·

THE HOMŒOPATHIC HOSPITAL, MELBOURNE.

blue drill relieved by a narrow fancy white stripe. It is a substantial material, and looks as if it ought to stand any amount of hard wear. The skirt is made full and turned up round the bottom with a deep hem. It is gathered into a band at the waist, to which the bodice is also attached. The bodice is quite plain and tight fitting, and buttons in front under a voluminous apron of white linen. A bib high up to the throat is worn ending in straps that cross behind and of the head nurse or sister, wears a neat uniform of narrow lilac and white striped point. It is very fresh and clean looking, and has the advantage of keeping its colour to the end, an important consideration in these days of chemicals and bleaching powder. The cap and apron are similar in shape and material to those worn by her colleague, and have the merit of being useful both in size and shape as well as being eminently becoming.

### Everybody's Opinion.

[Correspondence on all subjects is invited, but we cannot in any way be responsible for the opinions expressed by our correspondents. No communication can be entertained if the name and address of the correspondent is not given, or unless one side of the paper only is written on.]

### MEDICO-PSYCHOLOGICAL ASSOCIATION AND ITS NURSES.

NURSE L. S. JONES writes: I have spent over two years in Berry Wood Asylum, during which time I have been for nearly twelve months in the sick ward. I have attended lectures on elementary anatomy and physiology, first aid, sick nursing, and mental nursing, besides practical classes in bandaging, &c.; I have passed the three parts of the first examination (written, oral, and practical), but circumstances compel me to leave mental nursing before completing the course for the certificate here, for which three years are required. With an examination (written, oral, and practical) at the end of each year, I am sure that I have already worked as hard as many hospital nurses holding certificates, but am not deemed qualified yet to hold a certificate of mental nursing, and some hospital nurses think we are not on an equal footing with them. Is this fair? Can you please inform me whether, with my training and experience here (I leave early next month), I can be permitted to attend at one of the Medico-Psychological Association examinations and gain their certificate?

### SECOND CLASS NURSES.

"A CORRESPONDENT" writes: You suggested in your remarks on "Private Nurse's" excellent letter in last week's HOSPITAL that it might be well to have a lower grade or second class of nurses. This seems to me to smack strongly of retrogression to the "Gamp" period from which we have most happily emerged. Surely for a nurse who is concerned in matters of life and death, a "little knowledge" is a more "dangerous" thing than in others on whose decisions more trivial issues depend. The nurses you spoke of would do very well as you say in mild cases, but who can tell at the outset of an illness whether the case is going to be simple or complicated? There is much more in nursing than merely keeping patients clean and making their beds; there are many dangers that can only be avoided by those who know they exist, and recognise them before they have laid too deadly a hold upon the patient, and understand the nature of the disease in hand and many others as well, and this knowledge is only acquired by years of practical work and close observation. I think in these days, when doctors are making such rapid strides in scientific knowledge, it behoves every nurse to avail herself of the most thorough hospital training, to keep herself well abreast with the age she lives in, which can be done by such aid as is afforded by your valuable paper, and in so doing enlarge her sphere of usefulness.

### MR. HALL CAINE AS A CARACATURIST.

A "TRAINED NURSE" writes: May I say a word through your columns on behalf of myself and several friends in the nursing world, *re* Mr. Hall Caine's "Nursing Nightmare." The poor trained nurses have been having a pretty rough time of it lately at the hands of various authors; but this is almost the last straw. We should much like to know where Mr. Hall Caine got his information in respect to his brilliant description of the "operating theatre" at "Bartimæus's Hospital," and where he met his heroine, Glory Quayle, probationer. We are glad he caused "the great doctors and matrons" to leave the scene of revelry "before the students and nurses commenced their evolutions in shadowland,"—we can imagine their dignified departure. And then, the "Hospital Chaplain," yes—with a great stretch of imagination we can see him receiving "Glory-fied" (Quayle) probationers at "Martha's Vineyard" after their midnight carousals, we can almost hear his "noisy laughter," and note the "passing ward sister" looking placidly on. Poor deluded Mr. Hall Caine. Evidently he knows nothing of the life-saving work carried on by brave, unselfish men and women in these midnight hours; nothing of the work silently carried on "for His sake" by our "hospital chaplain." It would be a vast pity to wake Mr. Hall Caine out of his dream, to tell him something of our real life. Let him go on presenting to his readers misleading details and insulting (to the profession) illustrations about our doctors and nurses—let him dream on—but we ask in the name of goodness, that Mr. Hall Caine will leave our beloved, unselfish, hard-working chaplain out of his senseless and misleading caricatures.

"ANOTHER TRAINED NURSE" writes: May I, as a "trained nurse," express my thanks—and I am quite sure what I say will be endorsed by many others—to the Editor of THE HOSPITAL for again defending that much-abused creature. Perhaps, as I happen to be a district nurse, I might consider myself exempt from the scathing criticisms of Lady Priestley; but, as her opinion of the necessary qualifications for a district nurse appear to be about as vague as Mr. Hall Caine's knowledge of hospital life and discipline (to say nothing of his absolute ignorance of construction), I prefer to take my place amongst those for whom it appears that just now no words are too uncharitable. I hope the day is far distant when we shall resent being told of our faults—and, doubtless, they are legion—but I very much doubt if any good can come of "these tirades." How a "private nurse" could construe the Editor's remarks into approval of them I fail to see, and I thank him most warmly for them and for his indignant protest against Mr. Hall Caine's unwarrantable misconception. Surely, even a novelist's licence should have limits, and I trust the suggested apology will be forthcoming.

### LADY PRIESTLEY AND THE NURSES.

"AN OLD PRIVATE NURSE" writes: I quite agree that it would be as well if Lady Priestley and many others could change places with us for a time. They would very soon come to the conclusion that a private nurse's life is not an enviable one, especially when the patients' friends interfere, or a "treasure of a lady's maid" makes it her business to spy and tittle tattle, and cause no end of mischief by not strictly adhering to the truth. We ignore such things, but, nevertheless, it causes a good deal of unpleasantness. The nurse has to contend with almost daily reprimands concerning things of which she has not the slightest knowledge, and, of course, is not allowed to say a word in self-defence because she is "only the nurse," therefore has no right to contradict false statements made by the treasure of a maid. Besides these trivial matters, the nurse very often does not get a night's rest for months, and is also on duty most of the day. If only people could see us as we are, if they saw only half our hardships and trials in real life, "not as the authors of trashy novels paint us," I feel sure we should enjoy more public sympathy than we do at present. In regard to fees, two guineas per week are well earned. I may add that many private nurses belong to institutions where the salaries vary from £24 to £30 per annum. It is very seldom we have the chance of attending any place of amusement, or enjoying the pleasures of life. Ours is, indeed, a very hard and trying life.

"SYMPATHY" writes: As a private nurse I feel it my duty to endorse the opinion expretsed by "A Private Nurse" in your columns of lost week's issue. It is not the lot of all private nurses to have for their patients "eligible single gentlemen" whose quarters are in "public watering places." We are not unacquainted with the petty persecutions perpetrated by the type of woman represented by the correspondent who opened this controversy. I can only too heartily endorse the feelings expressed by "A Private Nurse" with regard to the sense of dread and fear which always repeats itself on our starting for a fresh case. Surely our little recreations may be allowed as a set-off against the great responsibility we have when being left, as we often are, with dying patients, to answer for their lives to doctors and friends. Personally I have known what it is to have very bad sleeping accommodation, no consideration whatever being shown, when the most comfortable sleeping quarters (if a night nurse is to do her duty efficiently) are an absolute necessity. I have also known what it is to be badly fed. We think we may claim to know how to distinguish between the natural anxiety of loving friends and the fussy interferences of ignorant incompetency. It may not be unfit to remember that our services are only called in when the gravity of the case has proved ordinary nursing to be insufficient. Lady Priestley's objection to our fee as being excessive would leave all ordinary nurses out of the profession, and reserve professional nursing only to ladies of means. I may add that those seeking aid, while willingly paying large fees to the specialist, should not hesitate to pay us, who have all the tediousness of the work, our very moderate fee. Surely, too, Lady Priestley has overlooked the wear and tear of this arduous work, accelerated by the broken rest during the early stages of the illness.

### LADY PRIESTLEY AND THE NURSES.

"Truth" writes: There are two points in Lady Priestley's bitter attack on the modern nurse with which I agree. The first is the fact of so many (frequently pretty and attractive) young nurses being sent to nurse gentlemen in their chambers. The danger lies particularly in their being social equals, and thus having so much in common together. On the other hand, where men would make allowances for women in the lower ranks of life for being willing to nurse them as a means of livelihood, they will look askance at a young lady for doing it. There is no need to go into further details on the subject—it is neither nice nor wise for young nurses to nurse young men, whether in their homes or in chambers, whether married or unmarried. There are many nurses over thirty-five who are private nursing. They should take all these cases, and not the young inexperienced ones. The second point is the classification of nurses—as you yourself have put it—the first and the second class, with the two different gradation of fees—the first to tally with the successful practitioner, and the second with the half-crown doctor; the first class ones to be employed in houses where they can afford her fees and accommodate her as she has been accustomed to live (for in all justice you must acknowledge that it is hard on a nurse whose father has kept a butler—as Lady Priestley suggests—to be sent to nurse a publican's wife, or a shoemaker's child, and to have to put up with their style and mode of living). Lady Priestley does not know what lady nurses have to put up with, otherwise she would show more sympathy with the girl who, having been brought up in a refined, cultured home, is knocked about from pillar to post to earn—two guineas a week (they are very bitterly earned sometimes). And all I can add is, God help every gentlewoman who is obliged to earn her living by private nursing under the "present" *régime*. It is these very depressing hardships which drive lady nurses into rushing in for any bits of amusement or enjoyment which present themselves to them. It is at these moments that really nice girls are driven into doing foolish and injudicious things, and thus unwittingly get the whole profession into disrepute. Let Lady Priestley have a run of lower middle class people to nurse for several months—to have their pictures and ornaments to gaze at, their conversation and jokes to laugh at, to set at table with them, eat their stodgy puddings and their substantial pies, to be patronised by them, to be introduced to all their friends (for the swagger of the thing) as being able to afford a superior 'ospital nurse—to hear of your own class talked of as "people 'igh up in the world." It is very funny to hear and read, but it is not funny to experience, and it needs a very strong mind and character when you get out of such an *entourage* not to relish to the utmost a more congenial atmosphere, and worse luck when it happens to be "young men," "chambers," and a *chaise longue*. I ask you, should this matter be considered or should it not? Perhaps if the public knew what some nurses *a la mode* have to put up with they would show them more sympathy or more Christian charity. Was there not a womanly woman amongst all those who saw the nurse in a *chaise longue* causing so much censure to take her aside and in a kindly, sisterly, or motherly way to give the girl a hint and bring her to her better self? In an editorial article in The Hospital a little while ago you spoke of chafing for a peerage for your colleagues. I, too, and many other nurses are chafing for a better position for our sisters—for the present we are *des déclassées*, only to be recognised as gentlewomen out of uniform and amongst our own friends. When we have means of our own we can afford to rise above it, but when we are poor and are obliged to work for our living we find it hard, bitter, and demoralising. Can you, as a gentleman, be chivalrous enough to understand us and help us get—not a peerage, but the position we were born in, that of "gentlewomen" though "nurses."

### THE R.B.N.A. AND MENTAL NURSES.

Miss Constance H. Andrew, 21, The College, Bromley, writes: As a trained certificated mental nurse, I have naturally followed with much interest the discussion as to the propriety of admitting mental nurses to registration by the R.B.N.A. While fully endorsing Miss B. Jones' remarks, I should like to draw attention to the fact that a similar course of training is insisted upon at St. Ann's Heath (Holloway Sanatorium Hospital for the Insane), Virginia Water. Although our course of training only extends over a period of two years, nevertheless within that space of time we pass through every grade of work, from the merely mechanical cleaning of a room to the fully responsible post of deputy charge nurse. While primarily devoting our energies to the patients' mental affections, we have to attend to their physical condition (a matter of great importance), and are frequently called upon to nurse them through surgical or medical illnesses, which are often associated with mental disease. The mere fact of our having an infirmary with accommodation for thirty or forty patients (in which each nurse must pass from three to six months) guarantees a fair amount of experience in general nursing. The above remarks are the outcome of my own experience, but, doubtless, there are many other institutions affording the same advantages to both nurses and patients, the particulars of which have not been brought under public notice. As regards registration, I feel sure that all those who take the trouble to study our methods of training will be convinced of our fitness to meet members of the R.B.NA. on an equal footing, and that the prejudice which at present exists to our admission to registration is, like so many of its class, born of ignorance. It should be remembered that the nursing certificate of the Medico-Psychological Association is given after examination both written and oral; that the paper for the written examination is set by the council of the association, and is the same for all candidates; and that the oral examination is conducted by a medical man of experience, who is not connected with the hospital or asylum in which the candidate has been trained. An examination conducted on such lines should command not less public confidence than the private examinations for certificates which are held by general hospitals.

### The Book World for Women and Nurses.

Our Baby: For Mothers and Nurses. By Mrs. Langton Hewer. Author of "Antiseptics: a Handbook for Nurses." Fourth edition, revised. (Simpkin, Marshall, Hamilton, Kent, and Co., 82, High Holborn, London).

We are pleased to see that Mrs. Langton Hewer's book for mothers and nurses, entitled "Our Baby," has reached its fourth edition. We quote from her introduction what seems a sensible suggestion, "That every girl should be obliged, before leaving school, to pass an examination on the subject of 'How to bring up a Baby.'" The subjects treated in the book are well arranged. We think that one chapter might well have been omitted—we allude to the chapter on deformities. Deformities are so serious that to get early advice from the surgeons is the only safe course to pursue. In the advice given as to the rearing of children, minute and clear instructions especially bearing upon the treatment of delicate children are given, which will prove of great value to mothers, who in these cases are often much perplexed as to what course to pursue. Should the child not thrive upon its food, or gain in weight as it should do, numerous alternative diets are suggested, among which a suitable one is likely to be discovered. At the end of the book are capital diet charts, and useful directions are given for the preparation of humanised and sterilised milks. The importance of a sufficient amount of sleep, which, next to proper feeding and warmth, is so necessary for young children, is also strongly impressed upon those who have the care of them. There is, lastly, a good chapter dealing with the ailments common to childhood, where directions are given for the preparation and use of disinfectants. We think that "Our Baby" is one of the best books of its kind that has been placed before the public.

### For Reading to the Sick.

### THE ROYAL ROAD TO HAPPINESS.

**Verses.**

O Lord, how happy should we be
If we could cast our care on Thee,
If we from self could rest;
And feel at heart that One above
In perfect wisdom, perfect love,
Is working for the best.

How far from this our daily life,
How oft disturbed by anxious strife,
By sudden wild alarms;
Oh, could we but relinquish all
Our earthly props, and simply fall
On Thine Almighty arms!

Could we but kneel and cast our load,
E'en while we pray, upon our God,
Then rise with lightened cheer;
Sure that the Father who is nigh
To still the famished raven's cry,
Will hear in that we fear.

Lord make these faithless hearts of ours
Such lessons learn from birds and flowers;
Make them from self to cease.
Leave all things to a Father's Will,
And taste, before Him, lying still,
E'en in affliction, peace.

**Reading.**

In Thy presence is fulness of joy; at Thy right hand there are pleasures for evermore.—*Psalm* xvi. 2.

O God of my salvation! teach me to rejoice; let me each day commence afresh the sum of Thy benefits, and may my soul forget not one of them.

In every gladness, Lord, Thou art the deeper joy behind. —*MacDonald.*

If there be a royal road to happiness, the simple-minded find it, and the peace and contentment they participate is a boon which the vexed and scheming adventurer, however well meaning, is seldom privileged to enjoy. There is only one way in which genuine simplicity can be attained by those who would fain reap its advantages.

Simple-mindedness is innocence transmuted into an active principle—ignorance of, and insensibility to, evil—manifesting itself by a single-hearted energy expending itself upon what is good.

Another form and fruit of single-mindedness is thoroughness. The character which is distinguished by simplicity of purpose suffers no loss of energy by the scattering of efforts. A steady gaze, an unwavering earnestness, a simple motive, will carry a man rapidly and pleasantly to his goal, and spare him many troubles by the way. Instead of being diverted by the distractions and misled by the so-called temptations that allure the loiterer through life, the simple-minded worker discharges his duty, and presses forward, unaffected by the hindrances which delay the less well-disposed travellers. Thoroughness is one of the priceless qualities of character and work. It enhances the value of every achievement, and gives tone to virtue. Many a bitter disappointment at the end of a career might have been avoided by thoroughness at the outset and on the way. Thoroughness secures a concentration of power, so that whatever has been accomplished, however small or great the sum of work may be, is characterised by completeness; and this, in itself, forms no inconsiderable element of perfection. The converse of thoroughness is purity; and this, too, is one of the striking characteristics of simple-mindedness. There is no admixture of false and true principles, because the heart is pure in its affections, and the mind clear and straightforward in its aims and purposes.—*J. Mortimer-Granville.*

### Minor Appointments.

Hull Sanatorium.—Miss M. Gray has been appointed Night Sister at this institution. She received her training at the King's Cross Fever Hospital, Dundee, and at Dundee and Perth Royal Infirmaries, having since held the position of night sister at the City Hospital, Little Bromwich, Birmingham.

Barton Regis Hospital, Eastville, Bristol.—Miss Georgina Williams has been appointed charge nurse of the women's ward at this institution. Miss Williams was trained at the Royal Portsmouth Hospital, where she worked for five and a-half years, taking charge duty after completing her training.

### Notes and Queries.

The contents of the Editor's Letter-box have now reached such unwieldy proportions that it has become necessary to establish a hard and fast rule regarding Answers to Correspondents. In future, all questions requiring replies will continue to be answered in this column without any fee. If an answer is required by letter, a fee of half-a-crown must be enclosed with the note containing the enquiry. We are always pleased to help our numerous correspondents to the fullest extent, and we can trust them to sympathise in the overwhelming amount of writing which makes the new rules a necessity. Every communication must be accompanied by the writer's name and address, otherwise it will receive no attention.

*Training in Midwifery.*

(119) Can I anywhere get training in midwifery in order to pass the L.O.S. examination by giving the time only (not more than six months)? *Anxious Nurse.*

Free training in midwifery is not to be had. Write to the Secretary Midwives' Institute, 12, Buckingham Street, Strand, enclosing stamped envelope for reply, and ask where you can get the necessary training for the least expense.

*Training.*

(120) Will you kindly tell me where a young girl under twenty-one could be received as probationer in fever or children's hospital? She would pay a premium or give her services for one or two years if required. —*F. B.*

Probationers are received sometimes as young as nineteen in children's hospitals. You will find a list of such institutions, with particulars of the premiums and qualifications required from candidates, in "How to Become a Nurse" (Scientific Press, 28 & 29, Southampton Street, Strand, London, W.C.) Write to Miss Lobb, Children's Nursing Home, Uplands, Loughton, Essex.

(121) I am 22 and want to train as a nurse. Please tell me the best course to take to get thoroughly trained in a London hospital or infirmary?—*E. E. H.*

Read the book recommended above. Most general hospitals do not take ordinary non-paying probationers under 24 or 25 years of age. At children's hospitals and at most Poor Law Infirmaries younger candidates are eligible. These particulars you will find in the above-mentioned book. Apply to the matrons for forms of application.

*Children's Nurses.*

(122) Can you tell me the address of a home for training children's nurses. I think in Holland Park, where a grey uniform is worn?—*Mrs. H.*

The address of the Norland Institute, where ladies are trained for children's nurses, is 29, Holland Park Avenue, W.

*Midwifery.*

(123) Can you tell me of any institute where a midwife (L.O.S. certificate) could get a month's practice for time given?—*L. M.*

Are you willing to pay for board and lodging during the month? If you will let us know this we will make inquiries for you.

*On Nursing Heart Cases.*

(124) "Poverty," who asks a question on this subject, is reminded that no anonymous queries can receive attention. Name and address for Editor's information must always accompany letters.

*Uniform.*

(125) Is there any reason why a nurse should not wear her uniform after her marriage?—*A. D.*

We should have thought "A. D.'s" own common sense, which, as a nurse, it is to be supposed she possesses, would have answered this query for her. Of course, if she is not engaged in any way in nursing, it is manifestly unfitting to continue to wear a distinctive dress. But once a trained nurse always a trained nurse, and if at any time she undertakes nursing work it is quite right and proper to again don a uniform to which by her past training she is entitled and which is alone suitable for attendance upon the sick.

*District Nursing.*

(126) Can you tell me where I can learn district nursing work by giving a short time to learn? I have had twelve months' infirmary training, and possess certificate for monthly nursing.—*London.*

Why do you not apply to the Queen Victoria Jubilee Institute, St. Katherine's Royal Hospital, Regent's Park, London, N.W., or to one of the local affiliated branches which train nurses for district work? You will find a complete list of such institutions in Burdett's "Hospitals and Charities" (Scientific Press, 28 & 29, Southampton Street, Strand, London, W.C.).

## Figures and Tables

**Figure f1:**